# Point of Care Ultrasound as a Diagnostic Tool to Detect Small Bowel Obstruction in the Emergency Department: A Case Report

**DOI:** 10.21980/J8XD1G

**Published:** 2021-04-19

**Authors:** Rebecca Nadelman, Jesper Aurup

**Affiliations:** * Rutgers Robert Wood Johnson Medical School, Department of Emergency Medicine, New Brunswick, NJ

## Abstract

Point of Care Ultrasound (POCUS) in the Emergency Department (ED) is a rapidly evolving field and has improved time to diagnose and management. POCUS of the bowel is still a novel concept for many departments. However, there is a potential application for POCUS to augment or replace traditional diagnostic imaging of small bowel obstruction (SBO). This is a case of SBO detected on POCUS in just a few minutes, which raises the possibility of POCUS improving time to surgical consultation and ED disposition in the future.

**Topics:**

Point of care ultrasound, small bowel obstruction, tanga sign.

**Figure f1-jetem-6-2-v1:**
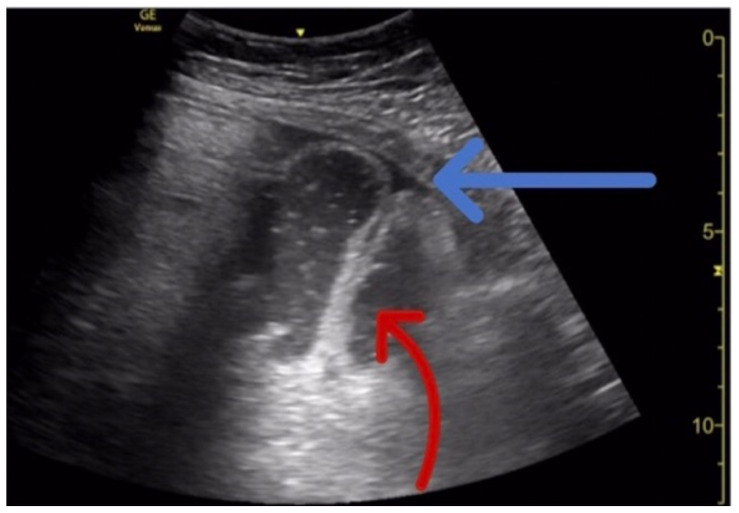
Video Link: https://youtu.be/TUunOoCtrp0[Fig f1-jetem-6-2-v1]

## Brief introduction

Small bowel obstruction (SBO) accounts for 15% of visits to the emergency department for abdominal pain, comprising 80% of intestinal obstruction.^1^ The most common cause in developed countries of SBO is adhesions from prior surgeries. As obstruction of the intestinal wall occurs, bowel distal to the site collapses and the proximal bowel dilates and fills with bowel contents, including air. Symptoms at presentation can vary, although classical presentations include diffuse colicky abdominal pain, nausea and vomiting, abdominal distention, and inability to pass gas. Complications include dehydration, ischemic bowel, as well as concurrent infection and sepsis. The differential for SBO is broad with many other emergent etiologies causing similar symptoms including but not limited to: mesenteric ischemia, AAA (abdominal aortic aneurysm), and perforated bowel. Therefore, the expedited diagnosis that point of care ultrasound (POCUS) allows helps to diagnose pathology efficiently.

The diagnosis of SBO has traditionally been achieved through the use of plain film abdominal radiographs or Computed tomography (CT), with CT having become the standard of care due to its ability to rule in or rule out other causes of the clinical syndrome and to pinpoint the location of the obstruction. Ultrasound offers several advantages over CT scan in the diagnosis of SBO. POCUS is inexpensive, non-radiating, non-invasive, and does not require the patient leaving the Emergency Department (ED) to diagnose SBO.^2^ In addition to these advantages, the expedited arrival to diagnosis improves ED flow and time to definitive care in cases where delays in SBO can occur secondary to the wait for CT imaging.

## Presenting concerns and clinical findings

A 65-year-old woman with a history of small bowel adenocarcinoma and recent admission for partial SBO presented with worsening nausea and abdominal distention as well as vague diffuse abdominal discomfort. She had poor appetite as well as several episodes of vomiting, and decreased flatus. Physical exam was remarkable for significant abdominal distention and diffuse tenderness.

This patient has a very high pretest probability of SBO while other diseases such as mesenteric infarction, AAA or perforated viscus were much lower in our differential diagnosis. Concerning findings for small bowel obstruction, in this patient’s case, included a history of malignancy and prior small bowel obstruction, decreased flatus, nausea and vomiting. The exam findings included abdominal distention and diffuse abdominal pain with palpation.

## Significant findings

The ultrasound findings suggestive of small bowel obstruction (SBO) are typically visualized in video; however, certain still images can also demonstrate SBO including greater than three dilated loops of small bowel (>2.5 cm), thickened-walled bowel (>3 mm), visualization of plicae circulares, and extraluminal fluid caused by inflammatory changes along the bowel wall, which are all highly suggestive of SBO.^3^

In this patient’s case, we were able to visualize several dilated loops of small bowel (red arrow) with oscillating intraluminal contents known as “Whirl Sign.” Additionally, we were able to visualize extraluminal fluid, demonstrated as an anechoic triangular-shaped collection. The characteristic shape of this triangular shaped collection of fluid is known as a “Tanga Sign,” given its name due to way it looks similar to the lower half of a bikini (blue arrow). Tanga sign can occur when the loops of dilated bowel appear prominent in contrast to the inflammatory extraluminal fluid in an SBO. These ultrasound findings were highly concerning for SBO which was later confirmed on CT imaging of the abdomen, which demonstrated SBO with a transition point in the left lower quadrant.

## Patient course

Upon initial evaluation of the patient, an intravenous line was placed and the patient was given medication to control her pain. Basic laboratory studies were ordered. POCUS of the abdomen was performed at the bedside. A curvilinear probe was utilized in an effort to visualize deeper structures in the abdominal cavity. POCUS revealed several dilated loops of small bowel with whirling intraluminal contents. The dilated loops were easily visualized with very little pressure applied by the examiner, and the patient was comfortable during the procedure. Surgery was consulted based on the ultrasound findings, but requested a CT scan of the abdomen and pelvis to still be performed, and a nasogastric tube (NGT) to be placed. The patient’s CT findings confirmed the initially suspected diagnosis on ultrasound and the patient was admitted to the surgical service for definitive management.

## Discussion

This case demonstrated the utility of point of care ultrasound in the diagnosis of small bowel obstruction (SBO) by visualizing an SBO in only a few minutes in contrast to a CT scan which takes much longer. The utility of Point of Care Ultrasound (POCUS) as a quick and easily accessible bedside tool to aid in the diagnosis of SBO could potentially allow the physician to diagnose an SBO more efficiently.

In cases of SBO, management generally centers around replenishment of fluids and appropriate correction of electrolyte abnormalities as well as bowel rest in consultation with the surgical team. If a patient has significant abdominal distention as in this case, gastrointestinal decompression can be achieved with an NGT.^2^ The need for gastrointestinal decompression in the setting of SBO may vary from patient to patient and remains a matter of clinical judgment.^3^ POCUS can potentially expedite this work-up in the emergency department as well as the final disposition of the patient. In this case, although a CT was ultimately requested by the surgeons, US showed the SBO in only a few minutes, while the patient had to wait much longer for the CT scan.

In contrast, abdominal CT scan with oral and intravenous contrast, the standard imaging modality in diagnosis of SBO,^4^ involves much more delay in care. Even the preferred imaging modality by the American College of Radiology Appropriateness Criteria of CT with intravenous contrast and no oral contrast is time-consuming when compared to ultrasound.^5^ While a bedside ultrasound takes a few minutes to perform, an abdominal CT can often take up to thirty minutes to take the images themselves.^6^ Besides the length of the procedure itself, a patient must drink oral contrast beforehand, which is often a very time-consuming process, especially if patients have significant nausea. Furthermore, the side effect profile of CT scan includes risk of radiation as well as immediate hypersensitivity reaction to contrast, which are spared with the use of an ultrasound.^7^

In a recent systematic review including 1178 patients, ultrasound was found to be 92.4% sensitive (95% CI [confidence interval] 89.0% to 94.7%) and 96.6% specific (95% CI 88.4% to 99.1%) with a positive likelihood ratio of 27.5 (95% CI 7.7%to 98.4%) and a negative likelihood ratio of 0.08 (95% CI 0.06% to 0.11%).^8^ For comparison, in a study examining the utility of CT scan in the diagnosis of SBO, the sensitivity and specificity of CT with oral as well as intravenous contrast were 91% (95% CI: 84%, 95%) and 89% (95% CI: 81%, 94%) respectively.^9^

There are several limitations to this case. As with any imaging modality, the use of point of care ultrasound in the diagnosis of SBO has some weaknesses. For example, detection of a distinct transition point on ultrasound is very difficult. Also, studies have demonstrated that while ultrasound displays high sensitivity for the diagnosis of SBO, in cases of partial obstruction it is not quite as sensitive.^10^ Sadly, as seen in this case and in our experience, most surgeons will still request a CT scan despite the ultrasound finding.

Point of care ultrasound offers promise as a tool to aid in the diagnosis of SBO. As compared to CT, ultrasound is a safer, easier, and more cost-effective imaging modality which can aid in earlier diagnosis and management of SBO. POCUS may also prove useful as a screening tool in patients with suspected SBO, much like an abdominal plain film. This may be helpful especially in areas where there may be limited access to CT. As evidence of the utility of POCUS in the medical literature increases, POCUS offers itself as a promising tool in the diagnosis of SBO in the future.

Note: The patient in this case gave verbal consent since non-identifiable images were taken. Only non radiologic images were used; therefore, written consent was not necessary.

## Supplementary Information






