# Supplementary figures and images for: Point of Care Ultrasound as a Diagnostic Tool to Detect Small Bowel Obstruction in the Emergency Department: A Case Report
Source: J Educ Teach Emerg Med. 2021 Apr 19;6(2):V1–4. doi: 10.21980/J8XD1G (PMC10332779; doi:10.21980/J8XD1G)

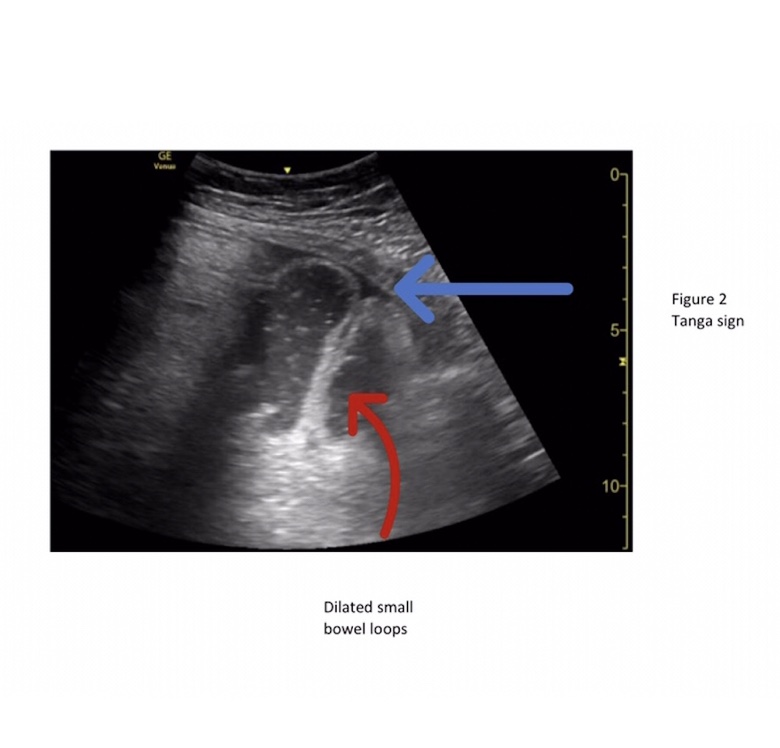

Supplement: Supplementary file 1 [file jetem-6-2-v1-supp1.jpeg]

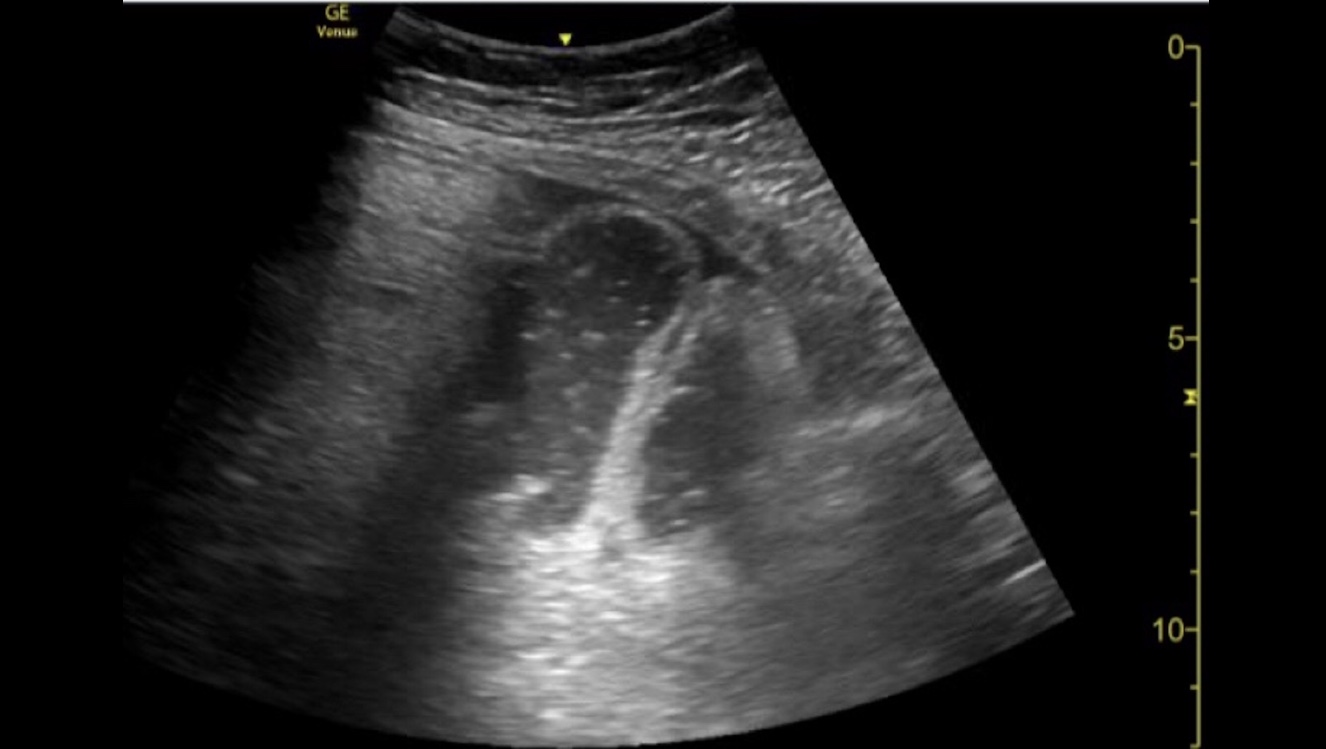

Supplement: Supplementary file 2 [file jetem-6-2-v1-supp2.jpeg]
